# Ausmaß und Entwicklung der Messerkriminalität in Deutschland: empirische Erkenntnisse und kriminalpolitische Implikationen

**DOI:** 10.1007/s11757-021-00692-7

**Published:** 2021-12-08

**Authors:** Elena Rausch, Whitney Hatton, Hauke Brettel, Martin Rettenberger

**Affiliations:** 1 Kriminologische Zentralstelle Wiesbaden (KrimZ), Wiesbaden, Deutschland; 2 Zentrum für Interdisziplinäre Forensik (ZiF), Johannes Gutenberg-Universität, Mainz, Deutschland; 3 Psychologisches Institut, Johannes Gutenberg-Universität, Mainz, Deutschland

**Keywords:** Messergewalt, Gewaltkriminalität, Staatsangehörigkeit, Schuldfähigkeit, Medien, Knife violence, Violent crime, Citizenship, Culpability, Media

## Abstract

Messerkriminalität steht seit einiger Zeit in der medialen und politischen Diskussion. Dabei wurde in der jüngeren Vergangenheit insbesondere über die Frage eines Anstiegs dieser Kriminalitätsform sowie über einen mutmaßlichen Zusammenhang mit der Staatsangehörigkeit der Täter/-innen diskutiert. Trotz der intensiven Debatten existieren bis heute vergleichsweise wenige gesicherte Daten hierzu. Der vorliegende Beitrag berichtet aktuelle empirische Ergebnisse über das Phänomen der Messerkriminalität anhand von Daten aus Rheinland-Pfalz. Ausgehend von einer Erhebung des rheinland-pfälzischen Ministeriums der Justiz wurden zu diesem Zweck Urteilstexte von insgesamt 519 rechtskräftig wegen schwerer Gewaltkriminalität abgeurteilten Personen ausgewertet, die sich auf Aburteilungen des Jahres 2013 (*n* = 253) und des Jahres 2018 (*n* = 266) beziehen. Die Ergebnisse zeigen, dass es keinen statistisch signifikanten Unterschied zwischen Messerkriminalität und schwerer Gewaltkriminalität insgesamt hinsichtlich der untersuchten Variablen, insbesondere der Staatsangehörigkeit, gibt. Auch ein massiver Anstieg der Messergewalt von 2013 auf 2018 konnte nicht nachgewiesen werden. Lediglich der Unterschied zwischen Messerkriminalität und genereller schwerer Gewaltkriminalität hinsichtlich der Schuldfähigkeitsbeurteilung war für beide Jahrgänge hochsignifikant. Die vorliegenden Ergebnisse lassen zusammenfassend deshalb nicht auf einen unmittelbaren kriminalpolitischen Handlungsbedarf schließen.

## Abstract (en)

Knife crime has been intensively discussed in Germany’s media and political debates in the last few years. Particularly the question of whether there has been a considerable increase in knife crime has been controversially debated but also whether there is a relationship with nationality and ethnicity. Despite these intensive discussions, there are comparatively little empirical data on this issue—at least in the German-speaking language area. The present article examined the phenomenon of knife crime based on data derived from the Federal State of Rhineland-Palatinate. For this purpose, the verdicts of a total of 519 persons convicted of a serious violent crime with final sentencing were included in the analyses, including convictions in the years 2013 (*n* = 253) and 2018 (*n* = 266). The data were compiled by the Ministry of Justice of Rhineland-Palatinate. The results showed no statistically significant differences between knife crime and severe violent crime overall regarding the studied variables, particularly nationality/ethnicity. Similarly, a massive increase in knife crime from 2013 to 2018 could not be found. Only the difference between knife crime and general severe violent crime in terms of culpability assessment was highly significant in 2013 and 2018. Therefore, the results of the present study could not serve as an indicator for an urgent need for immediate crime policy implications.

## Einleitung

In den vergangenen Jahren standen in Deutschland schwere Gewalttaten, bei denen ein Messer zum Einsatz kam und die auch als „Messerkriminalität“ bezeichnet werden, vermehrt im Fokus der medialen Berichterstattung. Dabei lag der Schwerpunkt der öffentlichen Debatten auf einzelnen besonders schwerwiegenden Gewalttaten; insbesondere wurde intensiv diskutiert, ob in Deutschland eine Zunahme derartiger Delikte zu verzeichnen war (*Tagesschau* 2020; *Focus online* 2019). Auch im (kriminal‑)politischen Diskurs wurde die Problematik aufgegriffen, wobei in diesem Kontext die Frage eines Anstiegs der (Messer‑)Kriminalität teilweise mit der Staatsangehörigkeit der Tatverdächtigen verknüpft wurde (z. B. *Landtag Rheinland-Pfalz* 2020). Ein zentraler Aspekt der Diskussion war dabei die Frage, ob die Zuwanderung von Asylsuchenden im Jahr 2015 einen Einfluss auf die Entwicklung der Kriminalitätszahlen hatte, wobei in diesem Zusammenhang zum Teil auch stigmatisierende und kontroverse Begriffe wie „Messermigranten“ oder „Massen- und Messereinwanderung“ verwendet wurden (Hestermann und Hoven 2019).

Der Raum für Spekulationen wurde auch dadurch vergrößert, dass in Deutschland das Messer als Tatmittel in der *Polizeilichen Kriminalstatistik *(PKS) nicht einheitlich gesondert erfasst wurde. Nur wenige Bundesländer führten bislang in ihren Kriminalstatistiken eine Auswertung, die sich speziell dem Tatmittel Messer widmet. Auch in der *PKS* des Bundes fehlte diese Kategorie bisher. Im Zuge der öffentlichen Debatte haben mittlerweile mehrere Bundesländer die Messerkriminalität in ihre *PKS* aufgenommen (*Focus online* 2019). Zukünftig soll auch die *PKS* des Bundes eine eigene Kategorie führen, die sich dem Tatmittel Messer widmet; mit einer umfassenden Umsetzung ist ab dem Jahr 2022 zu rechnen (*Deutscher Bundestag* 2021). Dabei definiert die *PKS* des Bundes Messerangriffe als „solche Tathandlungen, bei denen der Angriff mit einem Messer unmittelbar gegen eine Person angedroht oder ausgeführt wird. Das bloße Mitführen eines Messers reicht hingegen für eine Erfassung als Messerangriff nicht aus“ (*Bundeskriminalamt* 2020a). Um jedoch beurteilen zu können, ob tatsächlich ein Trend dahingehend vorliegt, dass die Messerkriminalität zunimmt, müssen die Häufigkeitsdaten über mehrere Jahre hinweg beobachtet werden (Neubacher 2020).

Um trotz der eingeschränkten Erfassungspraxis einen Eindruck von den Zahlen zur Messerkriminalität zu gewinnen, wurden in der Vergangenheit auf mediale und politische Anfragen hin Sonderauswertungen der polizeilich erfassten Daten durchgeführt (z. B. *Focus online* 2019; *Landtag Rheinland-Pfalz* 2020). Dabei ist jedoch zu beachten, dass diese Daten zunächst erhoben wurden, um die Ermittlungstätigkeit der Polizei zu unterstützen, und nicht für empirisch-quantitative Auswertungen. Überwiegend werden über die polizeilichen Systeme der Datenerfassung keine Informationen zu Ermittlungsverfahren protokolliert, weshalb die polizeilichen Erfassungssysteme im Hinblick auf eine Dokumentation der Kriminalitätswirklichkeit schon deshalb keinen Anspruch auf Vollständigkeit erheben (z. B. Landtag Rheinland-Pfalz 2020). Darüber hinaus erfassen diese Systeme Daten nach uneinheitlichen Kriterien, sodass die hieraus gewonnenen Zahlen nur bedingt vergleichbar sind (Heinz 2020). In Rheinland-Pfalz beispielsweise wurden die Daten dadurch erhoben, dass in dem *Geografischen Polizeilichen Informationssystem Kriminalität* (GeopolisK) nach Begriffen wie „Messer“, „Stichwaffe“ oder „stechen“ gesucht wurde. Auf diesem Weg ergab sich beispielsweise, dass im Jahr 2018 der Anteil der Körperverletzungs- und Tötungsdelikte, die mit dem Tatmittel „Messer“ und der Tatbegehungsweise „stechen“ registriert wurden, an der Gesamtzahl dieser Deliktskategorien 2,6 % betrug (*Landtag Rheinland-Pfalz* 2020).

Auch bei der Erfassung des Phänomens der Messergewalt in der *PKS* sollte beachtet werden, dass diese nicht justizförmig festgestellte Straftaten, sondern Fälle eines Anfangsverdachts erfasst und von Kriterien wie beispielsweise der Anzeigebereitschaft beeinflusst ist (Heinz 2020). Betrachtet man beispielhaft die *PKS* der *Polizei Berlin*, in der das Tatmittel Messer schon seit 2008 als Kategorie geführt wird, so ergab sich ein leichter Anstieg seit dem Jahr 2013 mit 2512 Fällen aus dem Bereich der Straftaten gegen das Leben, Sexual- oder Rohheitsdelikten hin zu 2675 Fällen im Jahr 2019^1^, was wiederum gegenüber den 2795 registrierten Fällen im Jahr 2018 einen leichten Rückgang bedeutet (*Landeskriminalamt Berlin* 2020). Zugleich zeigte die verstärkte politische Auseinandersetzung mit der Thematik bereits erste gesetzliche Auswirkungen: So wurde eine Änderung des Waffengesetzes beschlossen, die am 20.02.2020 in Kraft trat und es den Landesregierungen ermöglichte, die Einrichtung von Waffenverbotszonen auszuweiten, wobei ausdrücklich auf Messer Bezug genommen wurde (§ 42 Abs. 6 WaffG). Auch existieren bereits Präventionsprogramme, die explizit auf das Mitführen von Messern ausgerichtet wurden (Bartsch 2016).

### Empirische Fundierung

Neben solchen sicherheitspolitischen und kriminalpräventiven Maßnahmen gibt es in Deutschland bislang vergleichsweise wenige empirische Forschung, die speziell den Einsatz von Messern als Tatmittel in den Blick nimmt (Baier und Bergmann 2018; Thurnherr et al. 2008). Dies wurde teilweise damit begründet, dass es sich bei Messergewalt um ein relativ „alltägliches“ Gewaltphänomen handelt, weshalb hier die Forschung zu Gewaltkriminalität i. Allg. zum Tragen komme (Grimshaw und Ford 2018). Als zentralen Risikofaktor für den Einsatz von Messern sieht man das Mitführen eines Messers an, da hiermit ein gesteigertes Eskalationspotenzial einhergehe (Baier und Bergmann 2018; Emmert et al. 2018).

Von der deutschsprachigen Forschung wurde u. a. im Rahmen des *Niedersachsensurvey* des *Kriminologischen Forschungsinstituts Niedersachsen* (KFN) das Mitführen von Messern empirisch untersucht (zuletzt Krieg et al. 2020). Dabei zeigte sich, dass bei knapp der Hälfte aller Körperverletzungsdelikte mit Waffen Messer zum Einsatz kamen (Baier et al. 2018a). Trugen 2013 noch 16,9 % der befragten Schüler/-innen zumindest selten ein Messer in Schule oder Freizeit bei sich, waren es 2017 20,5 % (Baier et al. 2018a; Baier und Bergmann 2018), woraufhin 2019 ein (marginaler) Rückgang auf 19,3 % zu verzeichnen war (Krieg et al. 2020). Der zwischenzeitliche Anstieg ließ sich dabei v. a. auf das Verhalten männlicher Befragter zurückführen (Baier et al. 2018a; Baier und Bergmann 2018). In dieser Gruppe stieg die Anzahl derjenigen, die angaben, häufig ein Messer mitzuführen, von 8,5 % in 2013 auf 11,8 % in 2017 an. Damit führte 2017 fast jeder achte männliche Jugendliche in Niedersachsen häufig ein Messer mit sich. Zu Straftaten im Schulkontext unter Verwendung von Stichwaffen gab die *PKS* Rheinland-Pfalz Auskunft: Im Jahr 2019 wurde in 33 Fällen eine Stichwaffe mitgeführt; in 6 Fällen wurde diese auch eingesetzt (*Landeskriminalamt Rheinland-Pfalz* 2020).

Die Auswertungen des *Niedersachsensurvey* ließen auch auf mögliche Einflussfaktoren des Messertragens schließen (Baier et al. 2018a; Baier und Bergmann 2018). So wurde deutlich, dass Jugendliche, die in der Vergangenheit Opfer von Gewalt waren, möglicherweise zum Schutz vor wiederholter Viktimisierung häufiger ein Messer mitführten. Auch die Orientierung an gewaltlegitimierenden Männlichkeitsnormen wie Dominanz, Aggressivität und Wehrhaftigkeit sowie der Anschluss an eine delinquente Peergruppe spielten eine potenziell relevante Rolle. Ebenso hatte der Konsum illegaler Drogen einen möglichen Einfluss darauf, ob Jugendliche ein Messer mitführten.

Blickt man über die Grenzen Deutschlands hinaus, stand insbesondere in Großbritannien eine vergleichbare Problematik im Zentrum der öffentlichen Debatte (Brennan 2019; Skarlatidou et al. 2021), wobei sich dort die Forschung intensiver der Thematik annahm als hierzulande. Zunächst zeigten die vorliegenden Forschungsergebnisse, dass ähnliche Einflussfaktoren wie in der oben genannten Studie des *KFN* (z. B. vorherige Opfererfahrungen und Machtdemonstration) einen Einfluss auf die Wahrscheinlichkeit hatten, dass Jugendliche ein Messer mit sich führten (Harding 2020; Lemos 2004; Squires 2009). Ein wesentlicher Unterschied bestand hingegen darin, dass in Großbritannien der Fokus auf (Jugend‑)Gangs lag (Harding 2020; Kirchmaier et al. 2020; Skarlatidou et al. 2021), was wiederum in Deutschland ein eher untergeordnetes Phänomen darstellte. Die *Weltgesundheitsorganisation* (WHO) bezeichnete bereits 2010 Messergewalt als Problem in der Europäischen Union, wobei hier der Fokus auf jungen Akteuren im Alter zwischen 10 und 29 Jahren lag (*World Health Organization* 2010). Bemerkenswert in diesem Zusammenhang ist, dass Deutschland hier als eines der Länder mit der geringsten „Messertötungsrate“ genannt wurde.

Obwohl dem Thema Messergewalt folglich bereits länger eine erhöhte Aufmerksamkeit galt, wurde die öffentliche Debatte zumindest hierzulande lange Zeit auf der Grundlage einer vergleichsweise schmalen Datenbasis geführt. Das zentrale Ziel der vorliegenden Studie bestand deshalb darin, einen empirischen Beitrag zu dieser Diskussion zu leisten, indem Daten zur Häufigkeit der Messerkriminalität in Rheinland-Pfalz ausgewertet und diskutiert wurden. Dabei wurde das Phänomen der Messerkriminalität mit verschiedenen täter- und tatbezogenen Variablen in Verbindung gebracht und untersucht. Mit Rücksicht auf das dargelegte Empirie- und Theoriedefizit verzichtete man dabei auf die Formulierung konkreter (Unterschieds‑)Hypothesen und wählte ein weitgehend exploratives Vorgehen. Konkret untersuchte die Forschergruppe, ob es einen statistisch signifikanten Anstieg der Messerkriminalität unter den schweren Gewaltdelikten zwischen den Jahren 2013 und 2018 gab. Ebenso prüfte sie, ob im Hinblick auf das Geschlecht, die Staatsangehörigkeit und die Schuldfähigkeit – jeweils nach den Jahren 2013 und 2018 differenziert betrachtet – statistisch signifikante Unterschiede zwischen Messerkriminalität und der gesamten schweren Gewaltkriminalität bestanden. Auch war Gegenstand der Untersuchung, ob sich beim Vergleich der Aburteilungen^2^ aus den Jahren 2013 und 2018 statistisch relevante Unterschiede beim Altersmittelwert, dem Fehlen einer Täter-Opfer-Beziehung und einer Tatbegehung im öffentlichen Raum zeigten. Dabei wurden unter Messerkriminalität all jene Aburteilungen gefasst, bei denen es in mindestens einem Fall zu einer versuchten oder vollendeten Verletzung unter Messereinsatz oder der konkreten Drohung mit einem Messer gekommen war.^3^

## Methode

### Stichprobe

Die Untersuchungsgruppe bestand aus *n* = 519 Personen, die 2013 und 2018 rechtskräftig wegen mindestens eines Falls von schwerer Gewaltkriminalität in Rheinland-Pfalz abgeurteilt wurden (2013: 253 und 2018: 266). Sofern die Täter/-innen mehrere solcher Taten begangen hatten, wurden diese in einem Urteil zusammengefasst. Bei einer Person kam es nach dem ersten rechtskräftigen Urteil in 2018 erneut zu einem schweren Gewaltdelikt, für das sie noch im selben Jahr rechtskräftig abgeurteilt wurde. Eine weitere Person wurde sowohl 2013 als auch 2018 rechtskräftig wegen mindestens eines schweren Gewaltdelikts abgeurteilt. Detaillierte Angaben zur Deliktsverteilung bei den Aburteilungen enthält Tab. 1.

**Table 1:** 

	Häufigkeit schwerer Gewaltdelikte (*n* = 519)		Messerkriminalität (*n* = 66)	
	2013 (*n* = 253)	2018 (*n* = 266)	2013 (*n* = 27)	2018 (*n* = 39)
Widerstand gg. Vollstreckungsbeamte, *§ 113 Abs. 2 Nr. 1 StGB*	–	11	–	5
Sex. Übergriff/Nötigung/Vergewaltigung, *§ 177 Abs. 7 Nr. 1 und Abs. 8 Nr. 1 StGB*^*a*^	1	3	1	2
Mord, *§ 211 StGB*	8	13	5	9
Totschlag, *§ 212 StGB*	14	22	10	11
Gefährliche Körperverletzung, *§ 224 Abs. 1 Nr. 2 StGB*	56	76	12	20
Schwere Körperverletzung, *§ 226 StGB*	5	8	0	6
Körperverletzung mit Todesfolge, *§ 227 StGB*	2	–	–	–
Diebstahl mit Waffen, *§ 244 Abs. 1 Nr. 1 StGB*	109	130	3	1
Schwerer Raub, *§ 250 Abs. 1 Nr. 1 und Abs. 2 Nr. 1 StGB*	87	48	8	8

^a^Gesetzesreform 2016, vorher § 177 Abs. 3 Nr. 1 und Abs. 4 Nr. 1 StGB

Die Abgeurteiltengruppe bestand aus 471 Männern und 48 Frauen (2013: 233 zu 20; 2018: 238 zu 28). Das Alter der Abgeurteilten bei der letzten Tathandlung variierte 2013 zwischen 14 und 75 Jahren (*M* = 30,24; *SD* = 13,13) und 2018 zwischen 14 und 90 Jahren (*M* = 32,38; *SD* = 14,04). Davon betrafen 360 Aburteilungen Täter/-innen mit deutscher Staatsbürgerschaft (2013: 179 Personen und 2018: 181 Personen). Hinsichtlich der Schuldfähigkeitsbeurteilung wurde bei 61 rechtskräftig abgeurteilten Personen für mindestens eine der zugrunde liegenden Taten die verminderte Schuldfähigkeit gemäß § 21 StGB oder Schuldunfähigkeit nach § 20 StGB angenommen (2013: 21 und 2018: 40).

Insgesamt wurden 12,7 % (*n* = 66) der vorliegenden 519 Aburteilungen als Messerkriminalität klassifiziert (2013: 10,7 %, *n* = 27 und 2018: 14,7 %, *n* = 39). In 31 der rechtskräftigen Aburteilungen wurde mindestens eine der Messertaten im öffentlichen Raum begangen (2013: 14 und 2018: 17), bei 19 abgeurteilten Personen existierte in mindestens einem Fall keine dokumentierte Täter-Opfer-Beziehung vor der Tatbegehung (2013: 8 und 2018: 11). Weitere deskriptive Angaben der Stichprobe sind Tab. 2 zu entnehmen.

**Table 2:** 

	Häufigkeit schwerer Gewaltdelikte (*n* = 519)				Messerkriminalität (*n* = 66)			
	2013 (*n* = 253)	%	2018 (*n* = 266)	%	2013 (*n* = 27)	%	2018 (*n* = 39)	%
Männer	233	92,1	238	89,5	24	88,9	37	94,9
Frauen	20	7,9	28	10,5	3	11,1	2	5,1
Deutsche Staatsangehörigkeit	179	70,8	181	68	20	74,1	22	56,4
Vermindert/nicht schuldfähig	21	8,3	40	15	6	22,2	12	30,8
Keine Täter-Opfer-Beziehung	–	–	–	–	8	29,6	11	28,2
Öffentlicher Tatort	–	–	–	–	14	51,9	17	43,6
Alter in Jahren	30,24 (*SD* = 13,13)		32,38 (*SD* = 14,04)		34,04 (*SD* = 15,59)		34,62 (*SD* = 13,94)	

*SD* Standardabweichung

### Erhebungsmethode

Ausgangspunkt für die vorliegende Studie waren Daten, die das *rheinland-pfälzische Ministerium der Justiz*^4^ beim *Bundeszentralregister* zu Personen abgefragt hatte, die in Rheinland-Pfalz wegen Straftaten aus dem in Tab. 1 ausgewiesenen Katalog abgeurteilt worden waren. Anhand der im Katalog aufgeführten Straftatbestände wurden alldiejenigen Fälle erfasst, die der oben genannten Definition von „Messerkriminalität“ entsprechen. Auf Grundlage dieser Daten kontaktierte das *rheinland-pfälzische Ministerium der Justiz* die zuständigen Staatsanwaltschaften, die Informationen aus dem staatsanwaltschaftlichen Fachverfahren *web.sta* sowie die Urteilstexte zu den einzelnen Fällen übermittelten. Im Wege einer Dokumentanalyse glich die Forschergruppe dann in einer Nacherhebung die vorliegenden Daten mit den Urteilstexten ab und stellte gegebenenfalls Rückfragen an die beteiligten Staatsanwaltschaften. Darüber hinaus wurden zusätzlich Variablen anhand der Urteilstexte erhoben.

Erfasst wurden Merkmale der Gewaltkriminalität (Messerkriminalität, keine Messerkriminalität), des Geschlechts der abgeurteilten Person (männlich, weiblich) sowie deren Nationalität (deutsche vs. nichtdeutsche Staatsangehörigkeit), die Vortat-Täter-Opfer-Beziehung (Täter-Opfer-Beziehung, keine Täter-Opfer-Beziehung), Merkmale des Tatorts (öffentlicher vs. privater Raum) sowie die Beurteilung der Schuldfähigkeit (vermindert schuldfähig/schuldunfähig vs. schuldfähig). Darüber hinaus wurde das Alter zum Zeitpunkt der letzten Tathandlung erfasst. Kodiert wurde (dichotom) das Vorhandenseins bzw. Nichtvorhandenseins der Merkmale.

### Statistische Auswertungen

Die statistischen Analysen erfolgten unter Rückgriff auf die *SPSS*-Version 27. Zufallskritische Vergleiche der untersuchten Merkmale erfolgten in Abhängigkeit vom Skalenniveau des vorliegenden Datenmaterials. Das Alter der rechtskräftig abgeurteilten Personen wurde für die Jahre 2013 und 2018 mithilfe eines *t‑Tests *auf Mittelwerteunterschiede hin überprüft. Im Zuge der Signifikanztests wurde in 2 Fällen – der Zusammenhangsanalysen der kategorialen Variablen Geschlecht und Messerkriminalität für die Jahre 2013 und 2018 – der *Exakte Test nach Fisher* verwendet. Alle weiteren inferenzstatistischen Vergleiche erfolgten anhand von *χ*^*2*^*-Tests*. Zudem wurde für alle signifikanten Ergebnisse *Cramers V* als Effektstärkemaß berechnet, wobei *Cramers V *Werte im Bereich von 0 bis 1 annehmen kann und nach Cohen (1988) wie folgt zu interpretieren ist: Werte ab 0,1 entsprechen einem schwachen Effekt, Werte zwischen 0,3 und 0,5 entsprechen einem mittleren Effekt, und Werte ab 0,5 entsprechen einem starken Effekt. Inferenzstatistisch wurde die Ausprägung unterschiedlicher Variablen im Bereich der Messerkriminalität im Verhältnis zu ihrer jeweiligen Ausprägung im Bereich der gesamten schweren Gewaltkriminalität untersucht (Beispiel: Anteil der männlichen im jeweiligen Jahr rechtskräftig abgeurteilten Personen, die ein Messer verwendet haben, im Vergleich zum Anteil der männlichen im jeweiligen Jahr rechtskräftig abgeurteilten schweren Gewalttäter insgesamt).

## Ergebnisse

### Messerkriminalität 2013 und 2018

Im Jahr 2013 beinhalteten 10,7 % der rechtskräftigen Aburteilungen wegen schwerer Gewaltkriminalität mindestens einen Fall von Messerkriminalität, während es im Jahr 2018 14,7 % waren, wobei dieser Anstieg statistisch nicht signifikant war, χ^2^ _(df_ _=_ _1)_ = 1,86; *p* = 0,173.

Innerhalb der Personengruppe, die wegen mindestens eines Falls von Messerkriminalität rechtskräftig in den Jahren 2013 und 2018 abgeurteilt wurde, wurde zunächst ein möglicher Altersunterschied der Täter überprüft: Die 2013 rechtskräftig abgeurteilten Personen waren zum Zeitpunkt der letzten Tathandlung zwischen 15 und 75 Jahre alt (*M* = 34,04; *SD* = 15,59), während sie 2018 die Spanne von 16 bis 82 Jahre (*M* = 34,62; *SD* = 13,94) umfassten, wobei auch dieser Unterschied nicht die statistische Signifikanzgrenze erreichte, *t*_(df_ _=_ _64)_ = −0,16; *p* = 0,875.

Der Anteil der Täter/-innen, die wegen mindestens eines Falls von Messerkriminalität rechtskräftig abgeurteilt wurden, ohne vorhergehende Täter-Opfer-Beziehung, lag bei 28,8 % und unterschied sich kaum zwischen den Jahren 2013 mit 29,6 % und 2018 mit 28,2 %. Der Unterschied zeigte sich nicht als statistisch signifikant, χ^2^ _(df_ _=_ _1)_ = 0,02; *p* = 0,900.

Diejenigen rechtskräftigen Aburteilungen wegen Messerkriminalität, denen eine Tatbegehung im öffentlichen Raum zugrunde lag, machten 47 % der Fälle aus. Der Anteil ging dabei von 2013 mit 51,9 % auf 43,6 % in 2018 zurück. Der Unterschied wies ebenfalls keine statistische Signifikanz auf, χ^2^ _(df_ _=_ _1)_ = 0,44; *p* = 0,508.

Die beiden Gruppen gesamte schwere Gewaltkriminalität (*n* = 519) vs. Messerkriminalität (*n* = 66) bildeten den Ausgangspunkt für die Untersuchung der jeweiligen Variablen auf Relevanz im Folgenden. Dabei wurde inferenzstatistisch untersucht, inwieweit die Ausprägung eines Merkmals (z. B. Geschlecht, Staatsangehörigkeit) im Bereich der Messerkriminalität sich signifikant von der Ausprägung des Merkmals im Bereich der gesamten schweren Gewaltkriminalität unterscheidet.

### Geschlecht

Die Gruppe der gesamten schweren Gewalttäter/-innen (*n* = 519) bestand zu 90,8 % aus Männern, 2013 waren 92,1 % der rechtskräftig abgeurteilten Personen männlich und im Jahr 2018 89,5 %. Im Bereich der Messerkriminalität (*n* = 66) bestand die Täter/-innengruppe zu 92,4 % aus Männern (2013: 88,9 %, 2018: 94,9 %). Um zu prüfen, ob dieser Unterschied signifikant ist, wurden Signifikanztests jeweils für die beiden Erhebungszeitpunkte 2013 und 2018 berechnet. Da in beiden Fällen eine der erwarteten Zellhäufigkeiten kleiner als 5 war, wurde der *Exakte Test nach Fisher* verwendet: Weder für die 2013 rechtskräftig gewordenen Aburteilungen zeigte sich ein signifikanter Unterschied (*p* = 0,457) noch für die rechtskräftigen Aburteilungen des Jahres 2018 (*p* = 0,394).

### Staatsangehörigkeit

Des Weiteren wurde überprüft, inwieweit sich der Anteil derjenigen Personen, die wegen Messerkriminalität rechtskräftig abgeurteilt wurden, mit deutscher Staatsangehörigkeit über beide Erhebungszeitpunkte zum entsprechenden Anteil an der gesamten schweren Gewalttäter/-innengruppe unterschied. Innerhalb der gesamten schweren Gewalttäter/-innengruppe (*n* = 519) hatten 69,4 % der Täter/-innen die deutsche Staatsangehörigkeit, 2013 lag der Anteil bei 70,8 %, 2018 bei 68 %. In der Subgruppe der Messerkriminalität (*n* = 66) lag der Anteil der Personen mit deutscher Staatsangehörigkeit bei 63,6 % (2013: 74,1 %, 2018: 56,4 %). Im Folgenden wurde der Unterschied zwischen der Messerkriminalität und der gesamten schweren Gewaltkriminalität auf Signifikanz überprüft. Nachdem sich für beide Jahrgänge gemeinsam kein signifikanter Unterschied aufzeigen ließ, χ^2^ _(df_ _=_ _1)_ = 1,17; *p* = 0,280, wurde die Analyse zusätzlich getrennt für die Jahre 2013 und 2018 durchgeführt: Weder 2013, χ^2^ _(df_ _=_ _1)_ = 0,16; *p* = 0,688, noch 2018, χ^2^ _(df_ _=_ _1)_ = 2,85; *p* = 0,092, zeigte sich ein signifikanter Unterschied.

### Schuldfähigkeit

In der gesamten schweren Gewalttäter/innengruppe (*n* = 519) wurden 11,8 % als vermindert schuldfähig nach § 21 StGB oder als schuldunfähig nach § 20 StGB eingestuft; der Anteil stieg von 8,3 % in 2013 auf 15 % in 2018 an. In der Subgruppe der Messerkriminalität (*n* = 66) lag der entsprechende Anteil insgesamt bei 27,3 %, wobei ebenfalls ein Anstieg von 2013 mit 22,2 % auf 2018 mit 30,8 % zu verzeichnen war. Sowohl für die rechtskräftigen Aburteilungen aus dem Jahr 2013, χ^2^ _(df_ _=_ _1)_ = 7,70; *p* = 0,006, als auch für das Jahr 2018, χ^2^ _(df_ _=_ _1)_ = 8,85, *p* = 0,003, zeigte sich der Unterschied zwischen Messerkriminalität und gesamter schwerer Gewaltkriminalität als statistisch hochsignifikant. Die Effektstärke lag jeweils im schwachen Bereich (*Cramers V* = 0,174 bzw. *Cramers V* = 0,182).

## Diskussion

Ziel der Studie war es, empirische Daten über das Phänomen der Messerkriminalität bereitzustellen sowie diese inferenzstatistisch zu überprüfen und damit einen wissenschaftlichen Beitrag zur öffentlichen Diskussion zu leisten. Zusammenfassend konnte die vorliegende Untersuchung im Rahmen des öffentlichen Diskurses formulierte Annahmen (s. Abschn. „Einleitung“) zum größten Teil nicht bestätigen: Der leichte prozentuale Anstieg der Messerkriminalität von 10,7 % in 2013 auf 14,7 % in 2018 spiegelte keinen statistisch bedeutsamen Anstieg wider, womit die teilweise medial vertretene These eines massiven Anstiegs der Messerkriminalität (z. B. *Focus online* 2019) für Rheinland-Pfalz nicht gestützt werden konnte. Ohnehin stellt das Maß der medialen Berichterstattung, in welchem insbesondere die Gewaltkriminalität generell und zuletzt auch die Messerkriminalität stark repräsentiert sind, selbstverständlich keinen Indikator für das tatsächliche Kriminalitätsaufkommen dar (Gatzke 2017; Reuband 2001). Durch mediale Überrepräsentation kann aber ein verzerrtes Bild der tatsächlichen Kriminalitätslage entstehen, das zu übertriebenen oder unangemessenen Diskussionen und Reaktionen beitragen kann (Hess und Scheerer 1997; Kozlova 2013; Reuband 1998). Die Veränderungen beim Altersdurchschnitt der Täter/-innen waren statistisch nicht signifikant; insgesamt deuten die vorliegenden Daten darauf hin, dass die Messerkriminalität in Deutschland kein spezifisches Problem der Jugend zu sein scheint – wie beispielsweise in Großbritannien (Harding 2020) oder nach Berichten der *WHO* (2010) –, sondern in allen Alterskategorien auftritt.

Es konnte kein statistisch signifikanter Unterschied zwischen der Messerkriminalität und der gesamten schweren Gewaltkriminalität mit Blick auf das Geschlecht der abgeurteilten Person festgestellt werden. Männliche Täter sind insbesondere bei schwerer Gewaltkriminalität deutlich überrepräsentiert (Hermann 2009), sodass es kaum überrascht, dass auch bei der hier untersuchten Messerkriminalität 90,7 % der Täter/-innen männlich waren.

Weder in beiden Jahrgängen zusammengenommen noch bei einer separaten Analyse war ein statistisch signifikanter Unterschied zwischen der Messerkriminalität und der gesamten schweren Gewaltkriminalität hinsichtlich der Staatsangehörigkeit auszumachen. Deskriptiv zeigte sich hingegen ein Anstieg des Anteils nichtdeutscher Täter/-innen bei den Aburteilungen aufgrund von Messerkriminalität von 25,9 % in 2013 auf 43,6 % in 2018. Eine entscheidende Rolle könnte hierbei die Anzeigebereitschaft spielen, die beeinflusst, welche Taten ins Hellfeld gelangen (Hermann 2003), was z. B. bei Körperverletzungsdelikten laut Birkel et al. (2020) nur bei etwa einem Drittel der Fälle anzunehmen ist. Hierbei muss in Rechnung gestellt werden, dass ethnisch fremde Tatverdächtige ca. doppelt so oft angezeigt werden, im Vergleich zu Personen, die als Mitglieder der eigenen Ethnie eingestuft werden (Baier et al. 2018b; Walburg 2014), und dass die mediale Berichterstattung einen entscheidenden Einfluss auf die Kriminalitätsfurcht und damit auf das Anzeigeverhalten (Walter 2008) hat. Somit liegt nahe, dass eine Intensivierung der Berichterstattung auch die Anzeigebereitschaft erhöht, wobei Hestermann (2020) berichtete, dass nichtdeutsche Tatverdächtige ohnehin medial deutlich überrepräsentiert sind, was eine Verstärkung der Auswirkungen auf das Anzeigeverhalten und damit die Hellfelddaten erwarten lässt.

Die Zahl derjenigen Personen, die wegen Messerkriminalität rechtskräftig abgeurteilt wurden, und bei denen bei der zugrunde liegenden Tat keine Täter-Opfer-Beziehung vorlag, unterschied sich nicht statistisch signifikant zwischen den Jahren. Innerhalb der Jahre wiesen prozentual deutlich weniger Aburteilungen aufgrund von Messerkriminalität keinerlei Täter-Opfer-Beziehung auf (Tab. 2: 2013: 29,6 %, 2018: 28,2 %). Dies deckt sich mit dem kriminologischen Befund, dass Gewaltkriminalität überwiegend im sozialen Nahraum stattfindet (Gatzke 2017; Grafl 2013). Berücksichtigt werden muss bei den hier genannten Ergebnissen, dass auch ungeklärte Vorbeziehungen sowie Opfergruppen, bei denen ein beruflich bedingtes erheblicheres Risiko der Opferwerdung vorlag (Polizei, Sicherheitsmitarbeitende), als fehlende Täter-Opfer-Beziehung eingestuft wurden.

Auch hinsichtlich der Tatbegehung im öffentlichen Raum existierte kein Unterschied zwischen den Jahren, der über dem Zufallsniveau lag. Die Größenordnung – von 51,9 % in 2013 auf 43,6 % in 2018 – korrespondierte mit Befunden der *PKS*, wonach der Anteil der gefährlichen und schweren Körperverletzungen auf Straßen, Wegen, Plätzen 2019 bei 44,7 % lag (*Bundeskriminalamt* 2020b). Bemerkenswert ist, dass in der hiesigen Studie in über einem Drittel (38,7 %) der Fälle von Messerkriminalität im öffentlichen Raum eine relevante Täter-Opfer-Beziehung bestand, sodass das Risiko, zufällig im öffentlichen Raum Opfer eines Messerangriffs durch einen bis dato unbekannten Täter zu werden, als vergleichsweise gering eingestuft werden kann.

Ein statistisch bedeutsamer Unterschied zwischen der Messerkriminalität und der gesamten schweren Gewaltkriminalität zeigte sich für beide Jahre mit Blick auf das Vorliegen von Beeinträchtigungen der Schuldfähigkeit. Dieser Befund könnte damit zusammenhängen, dass bei Tötungsdelikten grundsätzlich die höchsten De- und Exkulpierungsquoten bestehen (Kröber und Dahle 2008). Als mögliche Gründe dafür werden der Ausnahmecharakter der Delikte, der eine psychische Störung nahelegen kann, Aggression als Resultat durch Suchtstoffe bedingter psychischer Störungen oder aber schlicht die höhere Gutachtendichte angesichts der zu erwartenden hohen Strafe diskutiert (Verrel 1995). Nimmt man diejenigen Aburteilungen aufgrund von Messerkriminalität, in welchen §§ 20, 21 StGB zum Tragen kamen, in den Blick, so lagen 14 von 18 Aburteilungen versuchte oder vollendete Tötungsdelikte zugrunde, sodass dieser Erklärungsansatz für die Einordnung der Ergebnisse der vorliegenden Studie relevant zu sein scheint.

### Kritik und Fazit

Zusammenfassend lässt sich feststellen, dass die Befunde zur Messerkriminalität in vielerlei Hinsicht nicht nennenswert von den Befunden zur sonstigen Gewaltkriminalität (Walter 2008) abweichen. Das Niveau von 10,7 % bzw. 14,7 %, auf dem sich die Messerkriminalität bewegt, stellt einen ernst zu nehmenden Anteil an der schweren Gewaltkriminalität dar, allerdings lieferte die hiesige Studie keine Anhaltspunkte für einen dramatischen Anstieg oder gar eine „Messer-Epidemie“ (*Tagesschau* 2020). Beachtet werden sollte gerade mit Blick auf die Rolle einzelner schwerer Gewalttaten mit Messereinsatz, dass solchen Taten stets eine große Aufmerksamkeit der Medien gilt und das dadurch entstehende Bild nicht unbedingt der Realität entsprechen muss (*Bundeskriminalamt* 2006; Hestermann 2018), aber dennoch (trotz offensichtlicher und einfach nachzuweisender Verzerrungen) einen starken Einfluss auf die öffentliche Meinung und die Kriminalpolitik haben kann (Bock 2019; Hess und Scheerer 1997).

Bei der Interpretation der Ergebnisse der vorliegenden Studie müssen methodische Beschränkungen berücksichtigt werden: Aufgrund der verfahrensbezogenen Herangehensweise stehen die Datenpunkte teilweise für eine Mehrheit von unterschiedlichen Tathandlungen, von denen mehrere auch die Kriterien der Messerkriminalität erfüllen konnten. Dadurch besteht die Möglichkeit eines Informationsverlustes, indem Einzelheiten bei den jeweiligen Taten ggf. nicht ausreichend berücksichtigt werden konnten. So wird der Datenpunkt beispielsweise bereits dann als „öffentlicher Raum“ gewertet, wenn eine der Messertaten dort stattgefunden hat, unabhängig von der Einordnung der anderen Tathandlungen. Der Anzahl rechtskräftiger Aburteilungen ist nicht zu entnehmen, wie viele Fälle der Messerkriminalität tatsächlich dahinterstehen. Aus solchen Gründen wäre eine weitere Differenzierung zwischen den verschiedenen Tathandlungen in zukünftigen Auswertungen wünschenswert.

Auch stehen vor einer rechtskräftigen Aburteilung unterschiedliche Filter (Bock 2019), sodass die hier vorliegenden Daten nur begrenzt mit denen der *PKS* oder anderer polizeilicher Erfassungssysteme vergleichbar sind. Die bei der hiesigen Studie gewählte Vorgehensweise bietet jedoch den Vorteil, mit gesicherten Erkenntnissen durch die Letztbewertung unabhängiger Gerichte über eine Datenbasis zu verfügen, die über den bloßen Verdacht hinausgeht. Zu beachten ist außerdem, dass den rechtskräftigen Aburteilungen unterschiedlich lange Verfahren vorausgingen. Zwar erstreckten sich sowohl 2013 als auch 2018 die Aburteilungen auf Tathandlungen, die maximal einen Zeitraum von bis zu etwa 7 Jahren zurücklagen. Jedoch ergibt sich eine potenziell unterschiedliche Streuung aller abgeurteilten Tathandlungen über diesen Zeitraum, sodass anhand der Daten keine vollständige Aussage über die im jeweiligen Jahr begangenen Delikte, sondern vielmehr über die erfolgten rechtskräftigen Aburteilungen getroffen wurde. Um ein genaueres Bild der Messerkriminalität zu erhalten, sollte zukünftige Forschung nicht nur ein Bundesland, sondern idealerweise das gesamte Bundesgebiet (oder zumindest mehrere Bundesländer) in den Blick nehmen, da hier aufgrund der geografischen Begrenzung nur eine relativ kleine Stichprobe untersucht werden konnte.

## Footnotes

1 Für den vorliegenden Artikel wurde bewusst auf *PKS*-Daten des Jahres 2019 zurückgegriffen und nicht auf die aktuelleren Zahlen aus 2020, da aufgrund der COVID-19-Pandemie von entsprechenden Verzerrungen der Kriminalstatistikzahlen auszugehen war.

2 Der Einfachheit halber wird im Folgenden von Aburteilungen gesprochen. Gemeint sind damit Verurteilungen, also auch Strafbefehle sowie Aburteilungen, in denen eine Maßregel angeordnet wurde (s. amtliche Begriffsdefinition des *Statistischen Bundesamtes*). In der Gesamtheit der Studie sind allerdings keine Freisprüche/Einstellungen enthalten, sondern ausschließlich Personen, gegen die eine Kriminalsanktion verhängt wurde.

3 Die im Folgenden berichteten Ergebnisse blieben nahezu unverändert, wenn die Drohung unter Verwendung eines Messers und der Einsatz eines Messers separat oder zusammengefasst analysiert wurden, weshalb aus Platzgründen eine gemeinsame Darstellung, zusammengefasst als „Messerkriminalität“, erfolgte.

4 Geringfügige Abweichungen zwischen den Ergebnissen, die das Ministerium der Justiz im Rahmen vorangegangener Veröffentlichungen berichtet hatte, und der vorliegenden Studienauswertung waren auf Übertragungsfehler zurückzuführen, die erst im Rahmen der gegenständlichen Datenanalyse sichtbar wurden.
